# Bilateral Adrenal Incidentalomas: A Case Report and Review of Diagnostic Challenges

**DOI:** 10.1155/2013/953052

**Published:** 2013-01-17

**Authors:** Anders L. Carlson, Annis M. Marney, Scott R. Anderson, Matthew P. Gilbert

**Affiliations:** ^1^Division of Endocrinology, Regions Hospital, University of Minnesota Medical School, 401 Phalen Boulevard, Saint Paul, MN 55130, USA; ^2^Division of Endocrinology, Diabetes and Metabolism, The University of Vermont College of Medicine, Burlington, VT 05401, USA; ^3^Department of Pathology and Laboratory Science, The University of Vermont College of Medicine, Burlington, VT 05401, USA

## Abstract

Incidentally discovered adrenal masses (incidentalomas) are common and present challenges both in diagnosis and management. When incidentally discovered adrenal masses are bilateral, a refined diagnostic approach is warranted since bilateral disease is more likely to be pathologic. We review a case of a 34-year-old man with incidentally discovered bilateral adrenal nodules. A comprehensive diagnostic strategy led to the diagnosis of bilateral pheochromocytoma caused by von Hippel-Lindau syndrome. He was successfully treated with bilateral laparoscopic adrenalectomy and has recovered well. While the initial diagnostic approach is similar to the unilateral incidentaloma, additional testing and/or genetic testing should be considered in the case of the bilateral adrenal mass.

## 1. Introduction

The incidentally found adrenal mass presents several complex management issues. Defined as a mass 1 cm or more in diameter found during a radiologic study done for reasons other than evaluation for adrenal disease, the adrenal “incidentaloma” is present in approximately 6% of the population [1]. The prevalence increases with age, with patients greater than 70 years old having a prevalence of 7%, compared to 0.2% for those age 20–29 [2]. Most are nonfunctional benign adenomas, and recently published guidelines address the optimal approach to these lesions [3]. However, the discovery of incidental bilateral adrenal masses requires special attention and an expanded diagnostic approach.

## 2. Case Presentation

The patient is a 34-year-old, Caucasian male who was referred to our endocrinology clinic for evaluation of resistant hypertension. The patient had been experiencing chronic headaches and intermittent episodes of chest pressure for approximately 6 months. He denied other associated symptoms including flushing, diaphoresis, and palpitations. There was no family history of endocrine neoplasms or early cerebral or cardiovascular disease. 

**Figure 1:**
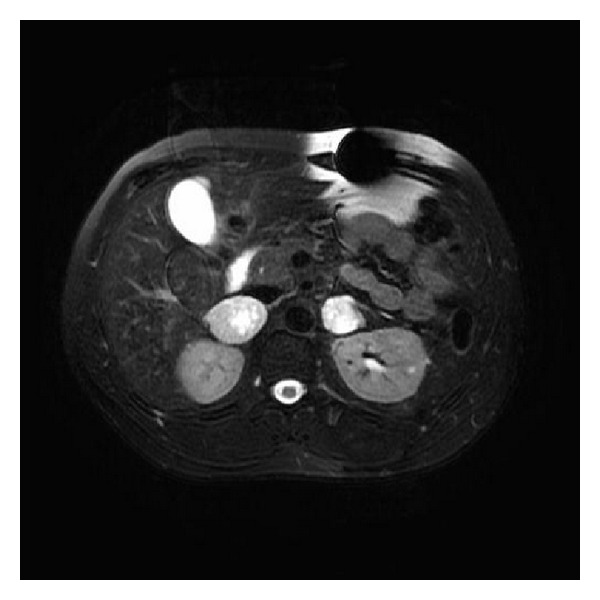
T2-weighted MRI of the abdomen showing bilateral adrenal masses. The left mass measures 4.3 cm and the right mass measures 2.8 cm in greatest dimension.

We obtained a renal artery duplex to evaluate for renovascular disease due to patient's young age and hypertension. The duplex demonstrated bilateral enlargement of the adrenal glands, and MRI of the abdomen showed high T2 intensity of bilateral adrenal masses with the right-sided mass measuring 4.3 cm and the left-sided mass measuring 2.8 cm (Figure 1). A 24-hour urine collection demonstrated a normetanephrine level of 9250 ug/24 hours (normal range 50–650 ug/24 hours). Plasma norepinephrine was also elevated at 3127 pg/mL (normal supine range 70–750 pg/mL) with a plasma normetanephrine of 23.1 nmol/L (normal < 0.90 nmol/L). Early morning ACTH and cortisol levels were normal. A plasma aldosterone concentration (PAC) to plasma renin activity (PRA) ratio was 2.8 (normal < 25.0). 

**Figure 2:**
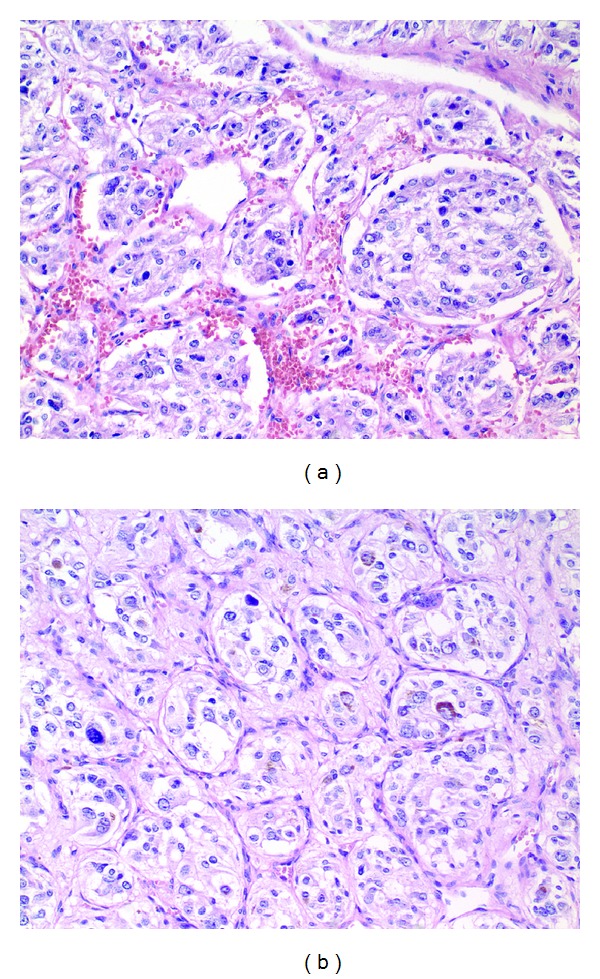
Histologic sections from the right (a) and left (b) show an alveolar (zellballen) architecture with tumor cells surrounded by a delicate fibrous framework. The tumor cells show variable nuclear pleomorphism and contain granular and basophilic to amphophilic cytoplasm. Hemorrhage (a) and hemosiderin (b) are common in these tumors. Both images are at 200x magnification and are stained with hematoxylin and eosin.

He was diagnosed with bilateral pheochromocytoma. The patient was started on phenoxybenzamine 10 mg twice daily which was titrated up to 60 mg twice daily; propranolol 40 mg twice daily was added once appropriate alpha blockade was achieved. Metyrosine was added prior to surgery. He underwent successful laparoscopic bilateral adrenalectomy, and the histopathologic features were consistent with pheochromocytoma (Figure 2). Staining for synaptophysin, chromogranin A, and S-100 protein were all positive. Subsequent referral for genetic testing and sequencing of the von Hippel-Lindau gene showed a deleterious mutation (R167Q). Additional genetic testing was not performed. No hemangioblastomas were seen on imaging, and he had no significant neurologic or ophthalmic findings. Following surgery, he recovered well and his hypertension has resolved.

## 3. Discussion

Approximately 15% of adrenal incidentalomas occur bilaterally [4]. Whereas most unilateral masses are benign or nonfunctional, the bilateral adrenal mass is more likely metastatic disease, hemorrhage, infiltrative disease, congenital adrenal hyperplasia, macronodular Cushing's syndrome, or bilateral cortical adenomas [5]. Bilateral pheochromocytomas are also possible, especially as part of a familial syndrome. The most likely primary cancers to metastasize to the adrenal glands are breast, lung, colon, kidney, and esophagus, though case reports exist for many other primary sites [6]. It is rare for metastatic disease to the adrenal glands to be the first manifestation of the primary cancer. Infiltration diseases, such as tuberculosis, histoplasmosis, and sarcoidosis, are also possible causes of bilateral disease.

In evaluating the hormone function of any adrenal incidentaloma, testing for subclinical Cushing's, pheochromocytoma, and hyperaldosteronism (in patients with hypertension) should be performed [1, 2]. A 1 mg overnight dexamethasone suppression test to screen for Cushing's syndrome and a 24-hour urine collection for fractionated metanephrines and catecholamines to screen for pheochromocytoma are recommended first-line tests [1]. Plasma aldosterone concentration (PAC) and renin activity levels (PRA) should be obtained and a PAC/PRA ratio calculated in patients with hypertension to screen for primary hyperaldosteronism. In the case of the bilateral adrenal incidentaloma, patients who present with symptoms such as hypotension, dizziness, weight loss, or abdominal pain in the setting of bilateral adrenal masses should be evaluated for adrenal insufficiency using an ACTH stimulation test (Table 1). Congenital adrenal hyperplasia (CAH) infrequently manifests as bilateral adrenal disease [7]. CAH is unlikely to present as an adult, since most patients are diagnosed in childhood or have symptoms of adrenal insufficiency, virilization, or salt-wasting [8]. If CAH is suspected, screening for adrenal insufficiency as well as mutation analysis for CYP21B is recommended. 

Specific attention should be paid to the unique clinical situation of the bilateral pheochromocytoma, which account for approximately 10% of pheochromocytomas [4, 5]. As in our case, where testing identified a familial etiology, most cases of bilateral pheochromocytoma are hereditary. Genetic syndromes of pheochromocytoma include multiple endocrine neoplasia type 2 (MEN2A and 2B), neurofibromatosis type 1 (NF1), the pheochromocytoma-paraganglioma syndrome (mutation of the *SDHB *or* SDHD* genes), and von Hippel-Lindau syndrome (VHL). MEN2A is a syndrome that includes hyperparathyroidism, medullary thyroid carcinoma, and pheochromocytoma, whereas MEN2B includes medullary thyroid carcinoma, pheochromocytoma, and mucosal neuromas. Neurofibromatosis type 1 is characterized by café-au-lait spots and neurofibromas. Mutations in the *SDHB* or *SDHD* genes predispose patients to glomus tumors and occasionally pheochromocytomas. Patients with bilateral pheochromocytoma should be screened for mutations in the genes associated with the above syndromes depending on the clinical presentation and family history (Table 1). If MEN2 is suspected, further testing with a calcitonin, intact PTH, and calcium level is warranted to evaluate for medullary thyroid cancer and hyperparathyroidism. Referral to a genetic counselor may be appropriate.

Von Hippel-Lindau syndrome is an autosomal-dominant syndrome characterized by cerebellar hemangioblastoma, renal cell carcinoma, retinal angiomatosis, and pheochromocytoma. Type 1 VHL uncommonly has pheochromocytoma. Type 2 VHL is either 2A (low risk for renal cell tumor), 2B (high risk for renal cell tumor), or 2C (pheochromocytoma alone). Our patient would fit most with VHL type 2C. The gene involved, *VHL*, is a tumor suppressor gene [9]. Pheochromocytoma is uncommon in VHL patients, with a prevalence of 14% in VHL kindreds [10]. They can be particularly challenging cases to manage because adrenal nodules may be present without symptoms of pheochromocytoma [11]. This likely occurs when the diagnosis of VHL is made before the clinical manifestations of the underlying pheochromocytoma are apparent. The discovery of an adrenal nodule in a VHL patient who has had a previous contralateral pheochromocytoma should be considered a pheochromocytoma until proven otherwise.

Definitive treatment for bilateral adrenal masses is surgical resection of both adrenal glands in most cases, except in the case of aldosterone secreting tumors, in which case medical options may be the preferred approach. Special care is needed in the case of the bilateral pheochromocytoma given the added morbidity of adrenal insufficiency after bilateral adrenalectomy. No specific guidelines exist addressing the optimal surgical approach to the bilateral pheochromocytoma; alpha-blockade followed by beta-blockade (with or without metyrosine) along with intravenous volume expansion remains the mainstay of preoperative management. Following bilateral adrenalectomy, care must be taken to ensure proper glucocorticoid and mineralocorticoid replacement.

## 4. Conclusion

The bilateral adrenal incidentaloma presents a unique diagnostic challenge. In addition to the risk of hormone hypersecretion, the bilateral adrenal mass carries additional risk of being metastatic from another primary carcinoma or part of a genetic syndrome. While the initial diagnostic approach is similar to the unilateral incidentaloma, additional testing and/or genetic testing should be considered in the case of the bilateral adrenal mass. Surgery remains the mainstay of treatment in most cases.

## Figures and Tables

**Table 1:** Recommended screening tests in adrenal incidentalomas. Additional analyses in bilateral incidentalomas listed below will depend on the clinical presentation and family history.

Recommended screening for all incidentalomas	Test
Cushing's syndrome	1 mg overnight dexamethasone suppression test
Pheochromocytoma	24-hour urine collection for fractionated metanephrines and catecholamines
Primary aldosteronism (screen only in hypertensive patients)	Plasma aldosterone to plasma renin activity ratio

Additional screening recommended for bilateral incidentalomas	Test

Adrenal insufficiency	Morning cortisol and ACTH (or corticotrophin stimulation test)
MEN2	*RET* gene mutation analysis, evaluation for hyperparathyroidism, medullary thyroid cancer, or mucosal neuromas
Von Hippel-Lindau syndrome	*VHL* gene mutation analysis and evaluation for additional tumors (such as renal, retinal, or nervous system)
Neurofibromatosis type 1	*NF1* gene mutation analysis
Pheochromocytoma-paraganglioma syndrome	*SDHB/SDHD* gene mutation analysis
